# *Aggregatibacter actinomycetemcomitans* as the Aetiological Cause of Rheumatoid Arthritis: What Are the Unsolved Puzzles?

**DOI:** 10.3390/toxins14010050

**Published:** 2022-01-11

**Authors:** Sung Cheng Looh, Zoey May Pheng Soo, Jia Jia Wong, Hok Chai Yam, Sook Khuan Chow, Jung Shan Hwang

**Affiliations:** 1 Department of Biotechnology, Faculty of Applied Sciences, UCSI University, Kuala Lumpur 56000, Malaysia; sungcheng323@gmail.com (S.C.L.); yamhc@ucsiuniversity.edu.my (H.C.Y.); 2 Department of Biological Sciences, School of Medical and Life Sciences, Sunway University, Bandar Sunway 47500, Malaysia; soo_zoey@hotmail.com (Z.M.P.S.); karenwongjiajia@gmail.com (J.J.W.); 3 Sunway Medical Centre, Bandar Sunway 47500, Malaysia; chowsk@sunmed.com.my; 4 Department of Medical Sciences, School of Medical and Life Sciences, Sunway University, Bandar Sunway 47500, Malaysia

**Keywords:** *Aggregatibacter actinomycetemcomitans*, leukotoxin A, hypercitrullination, rheumatoid arthritis, anti-citrullinated protein antibodies

## Abstract

Leukotoxin A (LtxA) is the major virulence factor of an oral bacterium known as *Aggregatibacter actinomycetemcomitans* (*Aa*). LtxA is associated with elevated levels of anti-citrullinated protein antibodies (ACPA) in rheumatoid arthritis (RA) patients. LtxA targets leukocytes and triggers an influx of extracellular calcium into cytosol. The current proposed model of LtxA-mediated hypercitrullination involves the dysregulated activation of peptidylarginine deiminase (PAD) enzymes to citrullinate proteins, the release of hypercitrullinated proteins through cell death, and the production of autoantigens recognized by ACPA. Although model-based evidence is yet to be established, its interaction with the host’s immune system sparked interest in the role of LtxA in RA. The first part of this review summarizes the current knowledge of *Aa* and LtxA. The next part highlights the findings of previous studies on the association of *Aa* or LtxA with RA aetiology. Finally, we discuss the unresolved aspects of the proposed link between LtxA of *Aa* and RA.

## 1. Introduction

### 1.1. Aggregatibacter Actinomycetemcomitans Is a Pathogenic Bacterium

*Aggregatibacter actinomycetemcomitans* (*Aa*) is a bacterium isolated from actinomycotic lesions of men and was termed more than a century ago by Klinger as ‘Bacterium actinomycetem comitans’ [1]. Its current scientific name has an interesting basis. *Aa* was, at the time of its debut as a novel bacterial species, co-isolated with the ray-shaped fungi *Actinomyces* spp, responsible for actinomycosis, which is a rare chronic fungal infection. Therefore, it was given the terms ‘actinomycetem’ and ‘comitans’, which can be literally translated as ‘ray-fungus– accompanying’. No taxonomic species or scientific name was given to this bacterium until 1929 when it was classified in a seminal textbook by Topley and Wilson under the genus *Actinobacillus* due to its morphological similarity with *Actinobacillus lignieresii* [2]. In 1985, *Aa* was reclassified under the genus *Haemophilus* based on the DNA–DNA hybridization results of a study that attempted to identify potential relationships between strains of *Actinobacillus* and *Haemophilus* genera [3]. However, this was objected to by Norskov-Lauritsen and Kilian [4] who assigned this bacterium to a novel genus called “*Aggregatibacter*”, an evolutionarily distinct branch of the *Pasteurellaceae* family, after citing their 16S RNA sequencing results. Since then, “*Aggregatibacter actinomycetemcomitans*”, which means “rod-shaped bacterium that aggregates with itself and other bacteria, and accompanies ray-fungus”, has been the bacterium’s widely accepted scientific name.

*Aa* is a Gram-negative coccobacillus that is 0.5–0.8 µm × 0.6–1.4 µm in size [5]. *Aa* is non-motile, non-spore-forming and does not form capsules [5]. Furthermore, it ferments fructose, glucose, maltose and mannose, although not sucrose, lactose, raffinose, melibiose, arabinose and trehalose [5]. *Aa* is also positive for catalase, oxidase, and H_2_S besides being negative for indole and ornithine decarboxylase. The bacterium reduces nitrate, and has lysine decarboxylases, and urease [6]. To date, there are 83 genome assemblies of different strains of *Aa* deposited in the NCBI database, of which only 12 are complete. The reference genome, encoding 1898 genes, is that of strain *VT1169* and has a size of 2.13 Mb (44.4% GC content). It is deposited under the accession number NZ_CP012958 [7]. Seven serotypes (a to g) of *Aa* have been identified based on the surface antigen O-polysaccharide side chain of their lipopolysaccharide [8,9]. Whether certain serotypes are more virulent than others is unclear, and their distribution in individuals suffering from periodontitis appears to vary across populations of different ethnicities [10].

*Aa* was once reported as the microbe that is most-commonly isolated from periodontal lesions. A total of 95% of patients with localized aggressive periodontitis were found to be infected by *Aa* [11]. This was especially true of the highly pathogenic serotype b clone, JP2, which was consistently isolated from Northwest African populations [12]. In this study, 60.4% of the 217 dental plaque samples obtained from periodontitis patients cultured positive for *Aa*. However, a longitudinal study conducted by Fine et al. [13], which examined 63 *Aa*-positive periodontally healthy individuals, appeared to contradict this finding when they found that more than 70% of their subjects remained healthy even three years after testing positive for *Aa*. Perhaps the discrepancies reported could be attributed to the differences in subject demography between the two studies since the association of *Aa* with periodontitis is more apparent in those of African or Middle Eastern descent [14]. Therefore, more work should be done in investigating the factors of susceptibility to periodontitis and its relationship with *Aa*. 

### 1.2. LtxA as a Major Virulence Factor of Aa

*Aa* has an arsenal of virulence factors, which allows itself to invade host tissues, evade the host immune system, and condition the host tissue environment for its survival. These virulence factors include adhesive factors, lipopolysaccharide (LPS), cytolethal distending toxins (CDT), outer membrane vesicles (OMVs), and outer membrane proteins (Omp). In this review, however, we focus on its intriguing toxin, leukotoxin A (LtxA) that specifically targets and kills leukocytes.

Leukotoxin A is considered the most significant virulence factor of *Aa* as it has been shown to be toxic to hematopoietic cells and therefore plays important roles in the evasion of host immunity and persistent infection in hosts. *Aa* extracts were first described as leukotoxic in a study by Tsai et al. [15]. Shortly after this discovery, the leukotoxicity was attributed to the LtxA isolated from *Aa* [15]. LtxA was also found to confer β-hemolysis capabilities to *Aa*, allowing it to lyse erythrocytes and release iron for its usage [16]. LtxA could be the triggering factor of pathogenic mechanisms in various human leukocytes, which contribute to the progression of periodontal diseases [17]. Periodontal disease is common and affects approximately 10–15% of the global population [18]. This disease is classified into two major groups, according to the periodontal disease classification system of the American Academy of Periodontology—(1) gingival disease, characterized by the inflammation of the gum tissue with no attachment loss of teeth, and (2) periodontitis, characterized by the formation of periodontal pockets caused by the loss of supporting connective tissues and alveolar bone around teeth. Within the umbrella of aggressive periodontitis, *Aa* is particularly associated with the localized form rather than the generalized form. Among various virulence factors produced by *Aa*, it is believed that LtxA is a key contributor to the pathogenesis of periodontitis. LtxA may kill leukocytes, thereby protecting *Aa* from phagocytosis and other host immune responses. The mechanisms, by which this occurs, could vary in different leukocyte types. Neutrophils, when exposed to LtxA, will degranulate and release proteolytic enzymes and metalloproteases, which contribute to periodontal tissue destruction and create a proteolytic environment that degrades immunoglobulins [19]. LtxA can also target macrophages and activate the inflammasome complex, which then leads to the secretion of pro-inflammatory cytokines. This intensifies inflammation at infected sites in patients with localized aggressive periodontitis, causing major discomfort [20]. Additionally, it has been found that neutrophils exposed to LtxA express citrullinated proteins characteristic of RA, implying the significance of LtxA as a major virulence factor in different diseases associated with *Aa* [21,22]. Due to its unique target specificity and complex actions that allow *Aa* to evade host immunity, LtxA has been studied intensively.

### 1.3. Ltx Operon

The leukotoxin operon of *Aa* encodes four genes, namely *ltxC*, *ltxA*, *ltxB* and *ltxD*. LtxA is encoded by the *ltxA* gene and consists of 1055 amino acids. The molecular structure of LtxA and its molecular interactions with its receptors are not well understood as its crystalline structure has not been resolved. Nonetheless, it is known that several clones of *Aa* express high levels of LtxA and are collectively known as the JP2 strains. These strains have a 530 base-pair (bp) deletion at the 3′ end of their promoter region, which has been shown to eliminate a transcriptional terminator of LtxA expression located at positions 298–397 of the 530 bp deletion [23].

Therefore, it is no surprise that JP2 clones have been reported to be highly associated with aggressive periodontitis, a rapidly progressing form of periodontal disease. This was supported by the prospective cohort studies conducted by Haubek et al. [24] and Höglund Åberg et al. [25], whereby a significant increase in the risk of aggressive periodontitis development was observed in individuals infected with highly leukotoxic (JP2) strains of *Aa,* however not in subjects infected with non-JP2 clones or low toxicity strains of *Aa*.

*ltxC* is responsible for fatty acid modification, which it has been suggested plays a role in the insertion of toxin into the lipid membrane of the host cell. Studies have shown an *ltxC* mutant was able to secrete similar LtxA levels as the wild-type strain, however this did not cause a calcium influx in the affected cells, suggesting that fatty acid modification is not required for LtxA secretion [26]. *ltxB* and *ltxD* are required for the secretion of LtxA and they are involved in the translocation and insertion of LtxA into the cell membrane of *E. coli* [27]. As for *Aa*, LtxB is predicted to be present in the inner membrane and associated with a predicted ATP-binding and hydrolysis site. This interacts with LtxD, which is attached to the periplasmic site of the inner membrane. 

### 1.4. LtxA Structure

**Figure 1:**
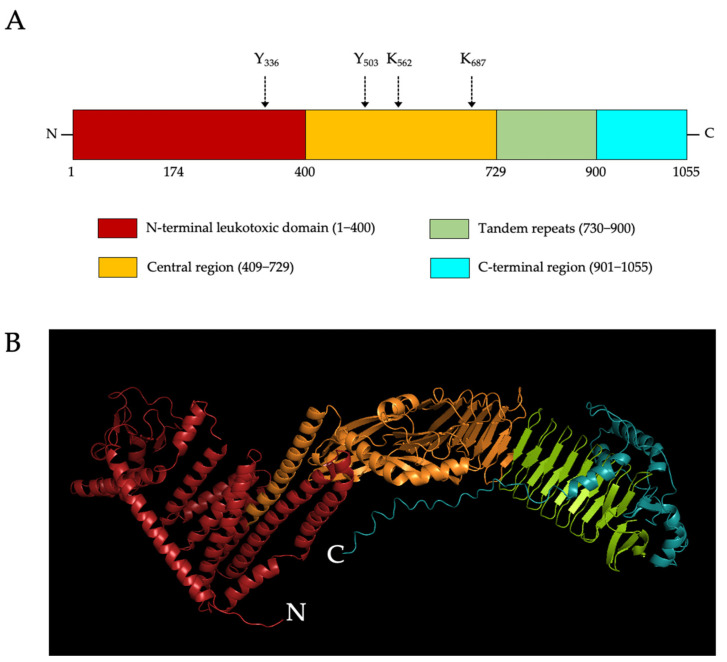
Leukotoxin A (LtxA) of *Aggregatibacter actinomycetemcomitans*. (**A**) Schematic drawing of the protein organization of LtxA adapted from Figure 3 in Lally et al. [28]. Each domain is indicated with different colors. The numbering below the domain structure refers to the amino acid positions. Arrows indicate the residues, which are important for cholesterol recognition and acylation sites. (**B**) The predicted tertiary model of LtxA (UniProt entry P16462) was downloaded from UniProtKB database and the predicted domains in (**A**) were colored using PyMOL version 2.5. Red, N-terminal leukotoxic domain; orange, central region; light green, tandem repeats; and cyan, C-terminal region.

The protein structure of LtxA has not been elucidated, but has been predicted based on the similarity of its DNA sequence to other genes of toxins belonging to the repeats-in-toxin (RTX) family [26]. LtxA is predicted to share approximately 51% sequence identity with the RTX toxin, *E. coli* alpha-hemolysin and approximately 43% identity with the *Mannheimia haemolytica* leukotoxin [26]. The toxin is estimated to be approximately 113 kDa in size [17]. Additionally, it is divided into 4 major regions (Figure 1), according to Lally et al., who described a widely accepted estimated structure of LtxA that is commonly used as a reference for current studies [28]. Residues 1–408 make up the N-terminal region, which consists of alternating hydrophobic and hydrophilic amino acid clusters. This region was shown through secondary structure prediction to be about 49% helical [28]. The leukotoxic properties have also been attributed to the hydrophobic clusters within residues 175–400 [28]. This N-terminal region is thought to be responsible for LtxA leukotoxic activity. It was also found that there is a cholesterol-recognition site in this region—tyrosine_336_, which contributes to the binding of the toxin to host cell membrane cholesterol, a key event of membrane association preceding target binding [29]. Residues 409–729, named the central region, is mostly hydrophilic with acylation sites at lysine_562_ and lysine_687,_ which may contribute to the anchorage of the toxin onto target cell membrane in a fashion similar to that of *E. coli* alpha-hemolysin [30,31]. This region also contains the other cholesterol recognition site, tyrosine_503_ [29]. Residues 730 to 900, making up the repeat region, consist of tandem repeats of nine amino acid cassettes with a common sequence of GGXGXDXUX, where X can be any amino acid, and U can be L, I, V, W, Y, or F [28]. In fact, there are 14 such repeats in LtxA that are collectively thought to be the binding site of the toxin to its target molecule, the lymphocyte function-associated antigen 1 (LFA-1), expressed on lymphocytes and other leukocytes [28]. The tandem repeats consisting mainly of glycine are also regions with a high binding affinity to Ca^2+^ ions, which mediate the increased binding of the toxin to LtxA-susceptible cells [32]. Residues 901–1055, termed the C-terminal region, are believed to be responsible for the translocation and secretion of the toxin since mutations in this region can cause the accumulation of LtxA in bacterial cytoplasm [28]. There are 20 more basic amino acids in this region of LtxA than in other RTX toxins, which results in the higher isoelectric point 9.7 of LtxA [33]. 

## 2. Hematopoietic Cells as a Target of LtxA

### 2.1. Leukocyte Function Associated Antigen-1 (LFA-1) as the Target Molecule

LtxA targets the LFA-1, a glycoprotein expressed on cells of hematopoietic origin [28]. LFA-1 mainly functions to mediate the migration of immune cells to the site of infection as it adheres to the intercellular adhesion molecule 1 (ICAM-1) expressed on vascular endothelial cells [34]. LFA-1, a heterodimer of integrins, is composed of the αL polypeptide chain CD11a and the β2 polypeptide chain CD18 [35]. Dileepan et al. demonstrated that LtxA is toxic to cells that express bovine CD11a and human CD18, although not to those expressing human CD11a and bovine CD18 [36]. This observation is likely as the extracellular region of human CD18 has cysteine-rich tandem repeats that encompass the integrin-epidermal growth-factor-like domains 2, 3 and 4 while bovine CD18 does not [36]. Kieba et al. demonstrated that CD11a has a 128-amino acid domain bearing its β propeller region, which is also important for LtxA-binding [37]. Interestingly, these two regions do not contain the required residues for interaction between LFA-1 and ICAM-1, indicating that LtxA likely interacts with LFA-1 in a different manner [38]. Nonetheless, the two regions of the two subunits are thought to only be available for binding when the LFA-1 molecule is in its activated or high-affinity state while carrying out its immune functions. At this point, the molecule will be in an extended conformation, which exposes the two regions mentioned [38]. As LFA-1 is activated by cytokines, which are released during an infection, such situations render LFA-1-expressing cells susceptible to LtxA. This makes LtxA a toxin that only attacks the most immunocompetent cells recruited to the site of infection [39]. The exact mechanism underlying the binding of LtxA and LFA-1 on target cells as well as the subsequent signalling pathways leading to cell death have not been elucidated. However, recent studies demonstrate that LtxA binds and immobilizes LFA-1, which eventually leads to LtxA internalization into the cytosol and the inhibition of integrin activation, ultimately leading to cell death [40].

### 2.2. Mechanism of LtxA-Induced Cell Death

**Figure 2:**
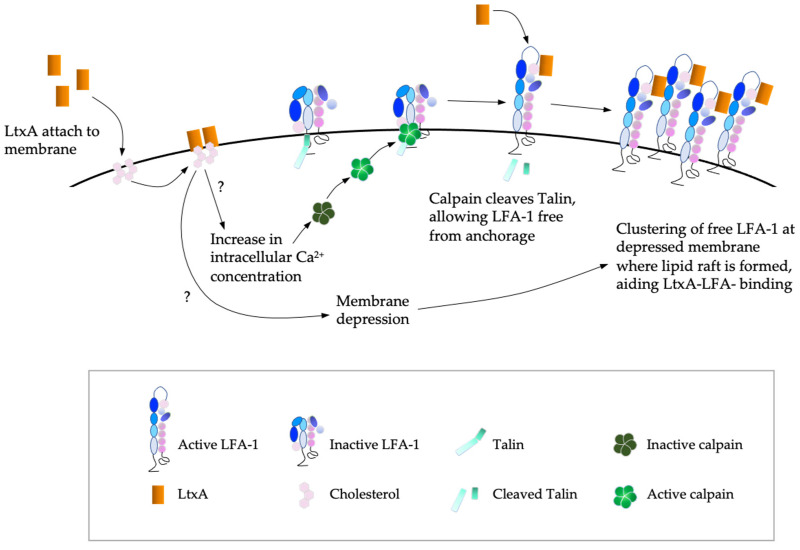
Illustration of the interactions of LtxA with cell membrane and its effects on target cells prior to LFA-1 binding. The question marks indicate signaling pathways, enzymes, or proteins, which have yet to be confirmed or identified as being involved in the process. Adapted from Figure 1 in Vega et al. (2019) [47].

Whether LtxA is truly a pore-forming toxin like other members of RTX family remains debatable as some reports found its pattern of membrane disruption different from that of pore-forming toxins [41]. Yet, many of the observed effects of LtxA on leukocytes coincide with the effects of the dysregulated influx of Ca^2+^ ions observed in cells treated with bacterial pore-forming toxins [42]. Despite its known specificity for hematopoietic cells through LFA-1 recognition, LtxA is found to first associate with the plasma membrane, inducing various changes in a non-specific manner. LtxA appears to bind to plasma membrane cholesterol using two cholesterol recognition sites located at its N-terminal region [29]. Apart from that, LtxA is also known to adsorb to the cell membrane via insertion of its fatty acyl chains, added during post-translational modification, into cell membranes. This is not observed in LtxA when the *ltxC* gene required for this acylation is deleted from its operon [43,44]. Once associated with the cell membrane, LtxA causes the formation of cell surface depressions followed by lipid-lined cholesterol-rich cavities or lipid rafts on the cell membrane (Figure 2) [41]. This destabilizes the membrane. Hence, at high doses, LtxA causes necrosis, which was believed to be the result of pore-formation some decades ago [45]. The currently accepted model of the pre-LFA-1 binding interactions of LtxA with host cell membrane involves an elevation of cytosolic Ca^2+^ concentration following membrane anchorage and adsorption. This then activates Ca^2+^-dependent intracellular cysteine protease, the calpain which cleaves talin, the protein holding LFA-1 to the cytoskeleton [43]. The clustering of talin-unbound LFA-1 at the lipid rafts where LtxA is adsorbed on the cell membrane appears to be the key event that causes LtxA-mediated cell death [46]. The exact mechanism of the cell death mediated by LtxA on LFA-1 expressing cells has not been established, however many different studies have provided insights that may help scientists solve this puzzle. Interestingly, the cell death pathways activated by LtxA appear to be different for each leukocyte type. The current knowledge on LtxA-induced lymphocyte, monocyte and macrophage death is not discussed in this review.

**Figure 3:**
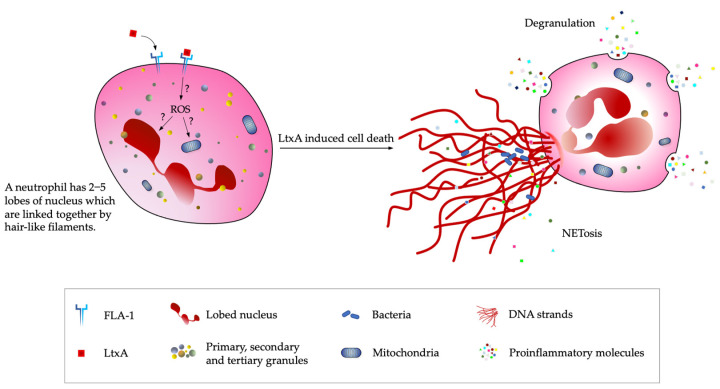
Schematic diagram of currently known LtxA-induced neutrophil death events. Upon LtxA binding to FLA-1 receptor on neutrophil, the release of cytochrome c from mitochondria is directly or indirectly caused by the reactive oxygen species (ROS) accumulated in the cytosol. The question marks indicate process/pathways unknown or unclear to date when ROS accumulated intracellularly. Apart from the mitochontrial-mediated apoptosis, degranulation and NETosis can be observed during the neutrophil cell death. Adapted from Figure 1 in Papayannopoulos (2018) [53].

Considering neutrophils are the most relevant leukocytes in both periodontitis and RA, LtxA-induced neutrophil death is discussed in this review. Limited information is known about the mechanism underlying LtxA-mediated neutrophil death as the lack of immortalized neutrophil cell lines poses a challenge to studies [46]. Nonetheless, under an electron microscope, neutrophils treated with LtxA were observed to show signs of degranulation and karyorrhexis [48,49]. Following degranulation, granule components, such as resistin, lysozymes, lactoferrin, neutrophil elastase and matrix metalloproteinase 8, are released to the extracellular environment [19,49,50]. This may explain the destruction of the periodontium of *Aa*-positive periodontitis individuals. Furthermore, it was found that these components are released before cell lysis based on the low level of extracellular lactate dehydrogenase during component release [46]. LtxA also causes a cellular process called NETosis, where neutrophils release web-like extracellular traps made of the aforementioned granule components and DNA strands into the extracellular space (Figure 3) [46]. NETosis is an innate defense strategy against bacterial products and infection, which is also observed in LtxA-treated neutrophils at a concentration of 10 ng/mL [21]. The exact mechanism and signaling pathways underlying the degranulation and NETosis of neutrophils after LtxA-LFA-1 binding are unclear. Whether the degranulation, karyorrhexis, and NETosis are events involving different LtxA-induced pathways is also unclear thus far. Peculiarly, at LtxA concentrations of 10 ng/mL to 100 ng/mL, only about 30% of neutrophils undergo NETosis, thus suggesting that LtxA-induced neutrophil death is not limited to NETosis but might also be dependent on signaling pathways that precede it, primarily reactive oxygen species (ROS) generation [21,51,52].

## 3. Insight into the Association between RA and Periodontitis

### 3.1. Previous Studies on the Association between RA and Periodontitis

The belief is that the periodontopathogens, their virulence factors and the immunocomplexes generated at the periodontal sites have the direct systemic access to the blood circulation and associate with various diseases such as atherosclerotic cardiovascular disease, diabetes, respiratory disease, chronic kidney disease, RA, Alzheimer’s disease and even cancer [54]. However, direct evidence for the causative role of periodontitis in these diseases is still lacking as there are many confounding factors that exist, such as age, gender and smoking. A large number of epidemiological studies and clinical studies have identified a strong relationship between periodontitis and RA. Table 1 shows the similarities shared between periodontitis and RA in epidemiology, pathophysiology, genetics, aetiology and molecular markers.

As both RA and periodontitis involve bone and tissue destruction, studies were conducted to investigate a possible association between these diseases in terms of their clinical parameters. The findings of certain studies indicated that there was an increased risk for periodontitis with RA. These include a study involving 50 RA subjects, which found this group to have higher incidence of gingival inflammation and bleeding, missing teeth, and increased probing depth (PD) and clinical attachment loss (CAL) [85]. Another study with 103 RA subjects found that this group was more likely to have periodontitis, regardless of age, sex, race or ethnicity, or smoking habit [86]. They were also more likely to have missing teeth than non-RA subjects. Additionally, rheumatoid factor (RF)-positive RA subjects were more likely to have periodontitis or tooth loss compared to RF-negative subjects, although this was not statistically significant. Havemose-Poulsen et al. [87] supported the finding that there were a significantly higher proportion of sites with periodontal bone loss, deeper pocket depths, and CAL values in RA subjects. They found a significant relationship between increased IgM-RF or IgA-RF and CAL. Another study noted significantly higher levels of CAL, PD, bleeding on probing (BOP), plaque index, and gingival inflammation in RA subjects [88]. RA subjects were shown to be 5.7 times more likely to have periodontitis and this likelihood increased to 8.05 times when the data was adjusted for age.

Nevertheless, most studies reported that the link between RA and periodontitis is likely bidirectional. Mercado et al. [89] found that RA was more prevalent in the periodontitis group and moderate to severe periodontitis was more prevalent in RA subjects. As such, 33 out of 36 of RA subjects under medication had evidence of moderate to severe periodontal bone loss. However, the study’s reliance on subjects’ self-reporting of RA was a major flaw. The same research group then conducted a follow-up clinical study involving 65 RA subjects and a 65 age- and gender-matched control group [90]. Comparing the RA and non-RA subjects, they found no statistically significant difference in the mean proportion of oral sites with plaques or gingival bleeding scores. However, there were more subjects with moderate to severe periodontal bone loss in the RA group and this group was twice as likely to have moderate to severe periodontal bone loss. RA subjects also tended to have more missing teeth. RA subjects with moderate to severe periodontitis had more categorically severe swollen joints, and higher serum CRP and erythrocytes sedimentation rate (ESR) levels. They discovered a significant association between functional debilitation and periodontitis severity, whereby 25% of RA subjects suffering from severe debilitation had moderate to severe periodontitis while those with less debilitation recorded no or mild periodontitis. It may be that it is harder for RA patients to maintain good oral hygiene practices as they may have reduced dexterity and functional ability. Kobayashi et al. [91] found a statistically significant positive correlation between RA disease activity levels and BOP rates in those with moderate to high disease activity, however they did not identify a significant association between disease severity and PD or CAL.

A study that only considered RA subjects, 147 in total, of which 83 had periodontitis, found a strong link between wrist destruction and periodontitis [92]. There was also a prominent relationship between HLA-DR shared epitope (SE) and bone destruction, whereby the former predisposed subjects to wrist and periodontal subjects by at least two times more. This indicates that SE is a major genetic factor of RA susceptibility and severity. 

Dissick et al. [93] studied the prevalence and severity of periodontitis in 69 US veterans with RA. A total of 35 veterans with osteoarthritis were selected as controls. Like previous studies, they found that RA subjects were more likely to have moderate to severe periodontitis, especially in the context of subjects positive for RF and ACPA. Multivariate assessment indicated that with each one-step increase in periodontitis severity, there was a 2.06 times likelihood of RA. Although the male-dominant population involved in this study may not accurately reflect reality, whereby women are more likely to be affected by RA, the statistical analysis conducted to draw inferences from the data had been adjusted for sex differences. Notably, to our knowledge, this was the first study to report the link between ACPA antibodies and periodontitis in RA context.

In fact, the high prevalence of periodontitis in RA patients as compared with non-RA control group had been reported in many other studies [87,88,90,93,94,95] and the same goes for the prevalence of RA in patients with periodontitis compared to non-periodontitis groups [55]. In addition, RA patients were found to be more likely to have moderate–severe periodontitis than non-RA subjects [96].

### 3.2. The Effect of Periodontal Therapy on RA

To further substantiate the link between periodontitis and RA, studies were carried out to investigate if treating the former would have any impact on RA disease severity. A study involving 40 RA patients found that periodontal treatment led to a significant decrease in their ESR, disease assessment scores of 28 joints (DAS28), swollen or tender joints, and improved patients’ assessment of their conditions [97]. Furthermore, 50% of patients that received periodontal treatment exclusively, and 40% of those who received both periodontal treatment and anti-TNF-α, demonstrated a marked decrease in serum TNF-α compared to 20% of controls. Another study consisting of 30 patients with moderate-to-high RA activity and 30 patients with low disease activity also found that periodontal treatment reduces disease severity in terms of DAS28, CRP, ESR, and TNF-α levels [98]. Meanwhile, in a case study, a 61-year-old RA patient showed improvement in joint pain and arthritis after periodontal treatment [99]. His DAS28 retreated to remission level while his ESR returned to normal. While his ACPA level remained high, he did not demonstrate increased severity. Zhao et al. [100] recruited 64 RA patients and divided them into four groups—those with both periodontitis and RA, those with neither, those with only RA, and those with only periodontitis. One month after periodontal treatment, patients with both diseases demonstrated significant reductions in CRP, ESR, DAS28, and ACPA. These studies had involved the same non-surgical periodontal therapies—scaling and root planning and oral hygiene instructions [97,98,99,100]. 

However, some studies using the same therapies yielded contradictory results. Pinho et al. (2009) conducted a study with 75 subjects: those with both periodontitis and RA; those with only periodontitis; those with only RA; and healthy controls. Their study found that, despite the fact that DAS28 levels were notably reduced at the time of the 3-month assessment, the periodontal treatment had no significant effect on the ESR and CRP levels of subjects with both periodontitis and RA [101]. Even more surprisingly, the ESR levels of untreated subjects with RA and periodontitis had decreased significantly by the 6-month assessment, while those of treated subjects had not. Another study, involving treatment (N = 20) and control (N = 20) groups, found that RA disease activity or SDAI significantly decreased eight weeks after the periodontal treatment [102]. This can be seen by the fact that the proportion of subjects with high RA disease activity was reduced from 61.1% of subjects to 27.8% of subjects. However, CRP, ACPA and RF levels were not significantly decreased as compared to controls. A study by Cosgarea et al. [103], which involved 18 subjects with RA and periodontitis and 18 subjects with only periodontitis, reported that only the CRP level was significantly reduced in RA and periodontitis subjects three months after the periodontal treatment. Other variable parameters such as RF, ESR and DAS28 of this treated group (RA and periodontitis) were not affected. The authors then explained that the CRP reduction at 3 months may merely reflect the resolution of periodontal inflammation rather than RA since there was also no decrease in the RF levels of the treated group. 

Taking all these studies together, although there are no conclusive results that indicate the effectiveness of periodontal treatment on RA activity, the consensus of the studies conducted is that periodontal treatment has a positive effect on RA severity, as reflected in some improved clinical assessments and parameters.

### 3.3. The Prevalence of Oral Microbiota Dysbiosis and Various Periodontal Bacteria in RA

In choosing to look beyond the correlation between the clinical parameters of RA and periodontitis, some studies assessed if oral microbiota dysbiosis is prevalent in those with RA. A study involving 31 new-onset RA, 34 chronic RA, and 18 healthy subjects conducted pyrosequencing to compare their subgingival microbiota composition [104]. Although there were no significant differences in the microbial richness or diversity between RA and non-RA subjects, it was found that periodontopathic bacteria are abundant in oral cavities of new-onset RA subjects, whereby *Leptotrichia* and *Prevotella* were characteristic species. *Porphyromonas gingivalis* (*Pg*) had no direct association with RA in this study as all three subject groups demonstrated high exposure prevalence to *Pg*, thus indicating it may be associated more with periodontitis.

Zhang et al. [105] investigated the gut and oral microbiomes of RA and healthy subjects to determine if there was any linkage between them. Using 212 fecal, 105 dental and 98 salivary samples, they found that RA had a strong effect on the salivary and dental microbiomes. Interestingly, Gram-negative bacteria such as *Haemophilus, Aggregatibacter, Cardiobacterium, Eikenella,* and *Kingella*, were abundant in the dental and salivary samples of healthy controls. *Pg* was also more abundant in healthy control samples here, which supports Scher et al. [104]. Meanwhile, anaerobes like *Cryptobacterium curtum, Atopobium* spp, and *Lactobacillus salivarius* were abundant in RA saliva and dental samples. Contrary to Scher et al. [104], *Prevotella* was found to be of pathogenic importance in this study, being positively correlated with RF.

A 2018 study involved 22 RA and 19 non-RA subjects who were periodontally healthy such that the direct effects of RA on the oral microbiome could be delineated [106]. RA subjects had oral microbiomes enriched in Gram-positive and Gram-negative obligate anaerobes compared to non-RA subjects. Meanwhile, facultative anaerobes were found to be more abundant in non-RA controls. The genera *Aggregatibacter*, *Gemella*, *Granulicatella*, *Haemophilus*, *Neisseria*, and *Streptococci* were less abundant in RA subjects, agreeing with Zhang et al. [105]. *Cryptobacterium curtum* was identified as a periodontopathogen of interest as it was dominant in the oral microbiome of RA subjects, giving rise to up to a 100-fold greater abundance in RA compared to controls. 

A study by Corrêa et al. [107], involving 42 RA and 47 non-RA controls, showed that RA was associated with greater microbial load and diversity. Additionally, its severity was linked significantly to the presence of periodontopathogens. Agreeing with Lopez-Oliva et al. [106], the study found that oral microbiomes of RA subjects were more abundant in Gram-negative anaerobes, likely responsible for periodontal destruction and inflammation, as seen in dental plaque samples. The findings here also showed that species like *Streptococcus, Kingella,* and *Haemophilus* were reduced in RA subjects, as discovered by Zhang et al. [105] and Lopez-Oliva et al. [106]. Meanwhile, the abundance of *Prevotella* was heightened in RA subjects. Interestingly, unlike other studies, *Aa* was significantly increased in RA subjects with periodontitis compared to non-RA subjects with periodontitis. However, as this study only involved a single timepoint, it could not determine if microbial dysbiosis is the trigger for, or the result of, RA. 

Numerous studies were carried out to pinpoint the prevalence of specific periodontal bacteria in RA based on titres of IgG antibodies against them and the presence of their genetic material in those with RA. Ogrendik et al. [108] and Okada et al. [109] found significantly higher levels of anti-*Pg* IgG in RA subjects compared to controls while Kimura et al. [110] identified similar titre levels between active and remission RA. The DNA of *Pg* has also been found to be prominently present in those with RA [103,111,112,113]. However, Scher et al. [104] did not find *Pg* to be prominent in RA subjects. As for *Eikenella corrodens* (*Ec*) and *Prevotella intermedia,* the titres of antibodies against them were not found to be significantly higher in RA subjects compared to healthy controls [109]. Titres against *Ec* were found to be higher in active RA than in remission RA, while titres against *Pi* were found to be higher in active RA and in synovitis-positive remission RA [108,110]. The presence of *Ec* genetic material was identified in RA synovial samples [112]. *Aa* was not found to be prominent in RA samples in terms of the levels of IgG antibodies against it and the presence of its DNA [103,109,110,111,112,113]. Yet, interestingly, Ebbers et al. [114] found that *Aa* sped up the onset of arthritis and its severity when inoculated into mice.

### 3.4. Protein Citrullination Bridges Periodontitis and RA

The clinical association between periodontitis and RA observed in multiple studies can be substantiated by a few common characteristics shared between the diseases. Of these, protein citrullination is of paramount importance. Since their discovery in 1964, ACPAs or anti-CCPs (anti-cyclic citrullinated peptides) have transformed RA diagnosis and the understanding of its pathophysiology such that tests for ACPA were incorporated into the 2010 American College of Rheumatology/European League Against Rheumatism diagnostic criteria [115,116].

ACPAs are specific biomarkers for RA that can appear years prior to the onset of symptoms. A 2004 longitudinal study found that a higher proportion of their sample RA population was positive for ACPA than for RF [117]. ACPA positivity emerged a median of 4.8 years earlier than symptom development whereas RF positivity had a median of 2.0 years. ACPA alone was also observed to be more sensitive than its counterpart and was associated with a higher risk of developing RA within five years. Interestingly, the subjects that demonstrated positivity for RF and/or ACPA presented greater frequencies of radiographic bone damage at the final follow-up. Another study with the same sample size supported these findings and noted that nearly half of its ACPA-positive subjects showed seroconversion to ACPA presence and antibody titres inclined approximately two to four years before diagnosis [118]. Two studies that investigated the association between ACPA and disease progression or outcome found that it was a predictor of RA severity, whereby it was linked with greater risk of radiographic damage and negative bone changes, respectively [119,120]. 

The association of ACPAs with periodontitis parameters or susceptibility in the context of RA cannot be discounted. In a population of subjects at-risk for RA, it was revealed that periodontitis was present in 100% of the ACPA-positive group and over 90% of them had moderate to high severity [121]. Meanwhile only 71% of ACPA-negative had mild to moderate periodontitis. Another investigation cemented the importance of ACPA in both diseases as it reported that significantly higher periodontitis values, such as CAL, plaque index, and PD, were found in the ACPA-positive group compared to the seronegative group [122]. There were significant correlations between these parameters and ACPA titres.

As citrullinated proteins are detected by ACPAs, it is important to illustrate that protein citrullination is a phenomenon common in both diseases. The occurrence of citrullination in RA was studied [123]. An abundance of citrullinated proteins were discovered in the synovial fluid of RA patients, making it an important site of expression. The mean levels of citrullinated α-enolase were elevated at least six times more in those with RA. Meanwhile, Nesse et al. [75] described citrullination in the oral stroma as inflammation-dependent and found citrullinated proteins in 80% of their periodontitis samples compared to 33% of their control samples. The study also reported that the presence of many prominent ACPA-targeting RA autoantigens in the periodontium, indicated that similar proteins might be citrullinated in RA- and periodontitis-affected tissues. Laugisch et al. [124] observed significantly higher baseline citrullination in non-RA patients with periodontitis than those without, while ACPA and anti-citrullinated enolase were elevated in RA patients regardless of periodontal condition. Anti-citrullinated fibrinogen antibody titres were higher in RA subjects compared to non-RA patients and amongst the latter the titres were higher in those with periodontitis than those without. Anti-citrullinated vimentin antibodies were also elevated in RA and non-RA subjects with periodontitis. Ultimately, these findings complement each other to suggest that citrullination is important in RA and, in the context of non-RA periodontitis patients, may lead to the violation of immunotolerance causing autoantibody production. 

Furthermore, Laugisch et al. [124] also showed that the citrullination of proteins, in turn, hinges on the expression and activity of PAD enzymes. The authors identified PAD activity in gingival crevicular fluid and noted an association between this and periodontitis. Their work was supported by Suzuki et al. [125]. In Suzuki et al.’s study, eight single-nucleotide polymorphisms, which had a significant association with RA on a chromosomal contig, contained *PADI4* genes. Besides confirming high PADI4 protein expression in haematological organs and cells, including polymorphonuclear leukocytes and bone marrow, they also identified the enzyme mRNA and citrullinated peptides in the synovial tissue sub-lining of all 7 RA subjects. This means *PADI4* is associated with high levels of antibody to citrullinated protein in RA subjects [125].

## 4. *Aa* as a Promising New Putative Periodontopathogen in RA Aetiology

Intrigued by the similarities in immune response and bone destruction between RA and periodontitis, Yoshida et al. [126] were the first group to assess this relationship by focusing on *Aa*. Following up from previous studies that suggested a possible role of heat-shock protein (HSP), DnaJ, from *Escherichia coli* in RA induction, they examined the presence of antibodies against *Aa* DnaJ in RA patients’ serum and the immunodominant region of this protein. The titres of IgG against the DnaJ1, J-domain and G/F region of *Aa* DnaJ were significantly higher in RA subjects compared to healthy controls. This indicated that *Aa* could be an interesting candidate for further studies.

In 2014, Romero et al. identified a pore-forming pathway that could lead to the intracellular activation of PAD enzymes and subsequent intracellular protein citrullination [127]. Granzyme B/perforin were identified as stimuli that led to significant cleavage of BH3-interacting domain death agonist (BID) and caspase-3. BID is a marker of extrinsic apoptosis. They uncovered that protein citrullination in RA patients spanned a wide range of molecular weights and this broad range citrullination was named “cellular hypercitrullination”. Granzyme B/perforin was the only extrinsic apoptotic stimulus, when compared to other RA-associated stimuli that could induce cellular hypercitrullination through caspase-3 activation. Upon further analysis, they discovered that perforin alone could induce this hypercitrullination and that activated PAD2 enzyme led to the most distinguished hypercitrullination. Perforin-activated PADs 3 and 4 were also found to induce hypercitrullination. Additionally, it was determined that the perforin pathway was calcium dependent. 

To further establish the involvement of pore-forming proteins in hypercitrullination, they assessed the possible role of complement proteins and found that neutrophil hypercitrullination relied on the presence of C7 proteins. Activation of the complement pathway results in the formation of a transmembrane pore-forming structure known as the membrane attack complex (MAC). The investigators then verified that the MAC/perforin pathway produces citrullinated autoantigens that closely mirror those found in RA synovial fluid cells, whereby the RA autoantigens overlapped completely with those in perforin-treated cells and significantly in the MAC-treated cells. Thus, the study put forth the roles of two immune-mediated pore-forming pathways that may be responsible for RA hypercitrullination.

This framework helped Konig and colleagues in their endeavor to explicate the underlying mechanisms of periodontopathogen-induced cellular hypercitrullination in neutrophils [22]. They discovered that the citrullinome of the RA gingival crevicular fluid had a similar pattern and spectra to that of the RA joint. Interestingly, they found that RA autoantigens only demonstrated citrullination of arginine residues within their polypeptides, which is characteristic of calcium-dependent human PADs, rather than at the C-terminals, which is characteristic of *Pg* PADs (PPAD). This argues against *Pg* as the candidate periodontopathogen and for other oral bacteria that can activate host PADs. 

To narrow down the possible periodontopathogens, they incubated neutrophils with various oral bacteria and found that only *Aa* could replicate the cellular hypercitrullination patterns observed in periodontitis GCF and RA synovial fluid. The findings of Romero et al. [127], which point to a pore-forming pathway for human PAD activation, and their own findings that hosted PAD-specific endocitrullination occurs in RA, led them to speculate that the LtxA of the *Aa* bacteria could drive PAD dysregulation and lead to cellular hypercitrullination. Indeed, they confirmed this theory as neutrophil hypercitrullination only occurred in the presence of purified LtxA and was abrogated in its absence. Additionally, they found that the LtxA-induced citrullinome overlapped with over 44 of the 86 proteins in the RA citrullinome, indicating that *Aa*-mediated neutrophil hypercitrullination in periodontitis may play a significant role in contributing to the autoantigens in ACPA-positive RA even though it may not be the sole inducer.

While previous studies attributed the release of citrullinated autoantigens to NETosis, this study found that LtxA-induced hypercitrullination was unaffected by neutrophil extracellular trap (NET) formation inhibitors. They also verified that LtxA-hypercitrullination is mediated by PAD enzymes as it was abrogated in the presence of PAD inhibitors. Furthermore, PAD activity was determined to be intracellular rather than extracellular and hypercitrullinated proteins were released with neutrophil chromatin. The findings obtained in this study using the various lines of inquiry affirmed the arguments put forth in a previous article by Konig and Andrade [128], whereby they had delineated a bacteria-associated phenomenon known as leukotoxic hypercitrullination (LTH). Among other characteristics, LTH was described as causing the citrullination of proteins across all molecular weights and ultimately, neutrophil death, which are two signature findings addressed in this review [22]. To strengthen the role of *Aa*, Konig et al. [22] also tested their hypothesis on clinical samples and reported that 41 of 196 RA subjects were positive for anti-*Aa* serotype b antibodies. There may be other serotypes of *Aa* that are involved. Anti-LtxA antibodies were also seen to be distinctively associated with RA. Contrary to previous studies, they found a strikingly high proportion of chronic periodontitis subjects with exposure to *Aa*, specifically its LtxA protein [108,109,110]. Anti-LtxA positivity in RA subjects was significantly associated with ACPA and RF levels, whereby ACPA of RA subjects positive for anti-LtxA antibodies primarily recognized the citrullinated autoantigens resulting from LtxA-induced hypercitrullination. This multi-faceted study offers persuasive evidence to implicate *Aa* as a candidate RA periodontopathogen as no other studies have identified other pathogens with the capacity to replicate the citrullinome spectrum in RA joints. 

Following the discoveries of Konig and colleagues, other research groups directed their efforts to investigating the role of *Aa* in RA. Laugisch et al. [124] also noted that *Aa* was significantly more prominent in the RA patient microbiomes than in non-RA microbiomes. Volkov et al. [129] reported that anti-LtxA antibodies were prevalent in a large proportion of RA subjects, although they were also prominent in other arthritides like osteoarthritis. They did not find ACPA-positivity to be limited to those positive for anti-LtxA antibodies. This does not contradict Konig and colleagues as they did not claim that ACPA positivity is exclusive to those with anti-LtxA antibodies. A 2018 case study reported the involvement of *Aa* infection in RA induction, where a patient infected with a highly leukotoxic strain of *Aa* developed severe RA symptoms [67]. However, after a course of antibiotics, the patient’s RA and autoimmunity-associated cytokine levels, and manifestations resolved progressively. This case offered direct evidence for *Aa* having an aetiological role in RA. Volkov et al. [129] attempted to replicate the findings of the Konig study in 594 RA subjects and found that anti-LtxA antibodies could be found in a significant proportion of RA subjects besides being present in other arthritides. However, this does not contradict Konig et al. [22] as they did not propose that anti-LtxA could only be exclusively found in those with RA. There was clarification that due to periodontitis being so common, anti-LtxA antibodies would be expected to be present in any individual with periodontitis, including those with or without other arthritides. 

A recent study examined the risk of developing RA after *Aa* exposure, which was delineated based on seroprevalence of anti-LtxA IgG, IgM and IgA [130]. Prior exposure to *Aa*, demonstrated by IgG or IgA positivity, was not found to be a risk factor for pre-symptomatic or diagnosed RA, although a portion of RA subjects had a significant increase in IgM, meaning that recent exposure may be linked with symptom onset. This was confirmed through a logistic regression analysis. The predictive capacity of anti-LtxA IgM positivity for early RA was found to be independent of other risk factors and each AU/mL of the antibody heightened the OR for RA development by 1.01. These studies collectively argue that the association between periodontitis and RA, and the role of *Aa* as an aetiologic periodontopathogen are worth investigating further. 

## 5. Can *Aa* and LtxA Unlock the Mystery behind the Triggering of RA?

Despite years of research, the exact aetiology of RA remains unknown, resulting in no cure for the disease or definite way to prevent it. Current treatments have mainly focused on achieving remission. An aspect that has been of increasing interest in RA aetiology research is the role of the oral microbiome. As we discussed above, the association between periodontitis and RA has brought attention to the aetiological roles of periodontopathogens, one of which is *Aa*. *Aa* secretes the virulence factor LtxA, which can trigger the cellular hypercitrullination in neutrophils, one crucial contribution to the breach of immunotolerance and autoantibody production in RA.

Although several RA studies have examined this bacterium, few have investigated its role extensively, and therefore questions still remain [22,124,126,127,128,129,130,131]. We address below the unresolved issues of *Aa*- or LtxA-mediated hypercitrullination in RA from the molecular and clinical perspectives. ACPAs are specific to RA and can be detected in 60–80% of RA patients [132], this group of RA patients is categorized as ACPA-positive. Only ACPA-positive RA appears to be associated with *Aa*-induced cellular hypercitrullination as based on the elevated level of anti-citrullination protein antibodies in sera. This suggests that there could be a different aetiology for ACPA-negative RA. The presence of ACPAs can be detected years before the clinical onset of RA [119], and is usually associated with more severe disease outcomes, such as more significant radiologic joint damage, compared to ACPA-negative RA [133,134,135]. There is uncertainty with regard to which underlying mechanism activates the autoimmune response in ACPA-negative RA. Moreover, it is unknown if ACPA-negative RA is periodontitis-related and if it could be caused by another type of periodontopathogen. As previously mentioned, the LtxA-induced phenomenon has been demonstrated to be consistent with leukotoxic hypercitrullination (LTH), a form of neutrophil death involving cellular hypercitrullination [128]. However, the mechanisms underlying LTH are still unknown as neutrophil proteins upregulated or downregulated by LtxA have not been identified. Molecular studies into this phenomenon are required to uncover its key proteins. Due to their role in citrullination, PAD enzymes are undoubtably one of the key proteins that one would expect to be upregulated. While PAD4 enzymes have been investigated for their role in RA citrullination more thoroughly, it remains to be seen if the hyperactivation of PAD2 enzymes have a direct role in cellular hypercitrullination [22,128]. Assessing its expression in neutrophils in response to LtxA will aid in establishing its importance in leukotoxic hypercitrullination. 

Additionally, it would be beneficial to further substantiate the role of leukotoxic hypercitrullination in RA by investigating whether the crucial effects of LtxA on neutrophils extend beyond the mere generation of citrullinated autoantigens. It is not known if LtxA, at certain concentrations, could upregulate certain neutrophil proteins or cytokines that serve to drive RA pathogenesis. For instance, B-cell activating factor (BAFF) and RANKL are cytokines produced by neutrophils that can stimulate the production of autoantibodies and osteoclast differentiation leading to bone resorption, respectively [136]. Determining if LtxA can encourage the production of these cytokines will give insight into the extent of its RA-inducing effects. 

About 400 bacterial species colonize in the gingival sulcus, the space between the tooth and its surrounding gum tissue, and they are predominantly the Gram-negative anaerobic bacteria (Table 2). Except those causing severe periodontal disease such as *Porphyromonas gingivalis, Treponema denticola* and *Aggregatibacter actinomycetemcomitans,* other subgingival bacteria have not been well-investigated in terms of their association with RA. Furthermore, studies that investigated the oral microbiota dysbiosis in RA did not divide their RA subjects into ACPA-positive and ACPA-negative individuals, which would have shed greater light on the differences of microbial diversity between the two categories or better pinpointed specific oral pathogens of interest. Perhaps in some studies, the importance of bacteria like *Aa* in RA could not be established since the data from these subjects were not analyzed as two distinct groups. Additional studies should be conducted where RA subjects recruited are equally divided into ACPA-positive and ACPA-negative groups, before being examined as two distinct groups to look for oral bacteria that may be crucial in ACPA-positive and ACPA-negative patients. 

Furthermore, it could be important to analyze the bacterial transcriptome in the gingival sulcus. The transcriptomic profile can be compared between RA groups only, those with periodontitis and RA, those with periodontitis only, and healthy subjects. The transcriptomes of these groups could vary significantly. 

*Aa* or LtxA may not be the sole pathogen or factor that could trigger ACPA production in RA, multiple factors may be involved in the same arthritogenic pathways of RA. They could be the causative factors or the risk factors. For example, the HLA-DRB1 allele, which lies within the human leukocyte antigen (HLA) class II region, is a risk factor for RA [137]. RA-associated HLA-DRB1 alleles have been reported to encode for a conserved amino acid sequence (QKRAA/QRRAA/RRRAA) comprising residues 70–74 in the third hypervariable region of DRβ1 chain. This conserved amino acid sequence, termed “shared-epitope (SE)” is located around the antigen-binding groove of HLA class II molecules, a site likely to affect the antigen presentation. It has been reported that exposure to *Aa* LtxA is strongly associated with ACPAs and RF in RA patients carrying HLA-DRB1 SE alleles [22]. Hence, there is a need to find out other causative or risk factors for RA.

## 6. Conclusions

RA is an autoimmune disease for which the cause has remained elusive. Without greater insight into its aetiological factors, improved therapies and a possible cure will be more difficult to achieve. Studies have shown that there is a link between periodontitis and RA, which is established by the activity of periodontopathogens. Researchers have highlighted that *Aggregatibacter actinomycetemcomitans* is one such candidate periodontopathogen due to the role of its LtxA in triggering neutrophil hypercitrullination. However, there remains an urgent gap in the knowledge of specific molecular pathways in neutrophils, which are affected by LtxA.

## Figures and Tables

**Table 1:** Summary of the features of periodontitis and rheumatoid arthritis.

No	Features	Periodontitis	Rheumatoid Arthritis
1	Prevalence	Affects about 20–50% of the global population [55], while its severe form affects about 10–15% of the global population [18]	Affects about 0.1–2.0 of the global population [56]
2	Modifiable/Environmental risk factors	Cigarette smoking; alcohol consumption; diabetes; obesity; stress; microorganisms	Regular smoking; diabetes; obesity; dietary; microbes at mucosal surfaces such as oral cavity, lung and gut [57]
3	Bone and tissue destruction	Alveolar bone resorption and bone loss in periodontitis patients [58]	Bone and cartilage break down [59]
4	Evidences supporting bacteria aetiology	High prevalence of anaerobic bacteria was found in the gingival tissues of patients with periodontitis [60]. Antibiotic treatment frequently results in improved clinical outcome [61,62,63]	Bacterial (including periodontopathogens) DNA and peptidoglycans are frequently detected in the synovial fluid and serum of RA patients [64,65,66]. Antibiotic treatment has been shown to effectively reduce RA disease activity [65,67]
5	Inflammatory markers	C-reactive protein (CRP), IL-1b, IL-6, TNF-a, visfatin, VEGF, oncostatin M, protein carbonyl, RANKL, IL-17, IL-36γ, MMPs and PGE2 [68,69,70,71,72]	CRP, IL-1b, IL-6, TNF-a, IL-23, IL-17A, IL-18, IFN-γ, PGE2, MMPs, RANKL and granulocyte macrophage colony-stimulating factor [73,74]
6	Autoantibodies	RA-associated autoantibodies eg. rheumatoid factors (RF) and ACPAs were present in the gingival crevicular fluid of non-RA periodontitis patients [75,76]. The positivity and titre of ACPAs are significantly associated with periodontitis [77,78,79] and alveolar bone loss [80]. Non-surgical periodontal treatment appears to reduce ACPA levels in periodontitis patients [81]	RF and ACPAs are prominently detected in the serum of RA patients. They are also associated with increased radiographic progression and joint damage [82]. Recently, anti-carbamylated protein antibodies (anti-CarP) and anti-acetylated protein antibodies were discovered in the serum of RA patients [83,84]
7	Citrullinated autoantigens	Histone H1x, adenylyl cyclase-associated protein 1, actin (cytoplasmic ½), apolipoprotein A-1 preproprotein, elongation factor 1-alpha, heterogeneous nuclear ribonucleoprotein A2/B1, histone H2A, vimentin, myeloid cell nuclear differentiation antigen, histone H2B were found in the gingival crevicular fluid of periodontitis patients [22]	Histone H1x, actin (cytoplasmic ½), apolipoprotein A-1 preproprotein, elongation factor 1-alpha, heterogeneous nuclear ribonucleoprotein A2/B1, histone H2A, vimentin, myeloid cell nuclear differentiation antigen, histone H2B were found in the synovial fluid of RA patients [22]

**Table 2:** Summary of different complexes of anaerobic bacteria species associated with periodontal health according to Socransky’s Classification.

Socransky’s Classification ^a^	Example	Remark
Yellow	*Streptococci sanguinis* and *Streptococci oralis*	Early colonisers essential for colonization of other bacteria associated with periodontal disease
Green	*Capnocytophaga* spp., *Campylobacter concisus, Eikenella corrodens*, and *Actinobacillus actinomycetemcomitans*	
Violet	*Actinomyces odontolyticus* and *Veillonella parvula*	
Orange	*Prevotella intermedia, Prevotella nigrescens, Micromonas micros, Fusobacterium periodonticum, Eubacterium nodatum* and *Streptococcus constellatus*	Bridging colonizers that aggregate with early and later colonizing bacteria
Red	*Porphyromonas gingivalis, Treponema denticola* and *Tannerella forsythia*	Late-colonizing bacteria, which are strongly associated with periodontitis and co-exist with the orange complex.
Ungrouped	*Aggregatibacter actinomycetemcomitans, Selenomonas*	More research is needed to group these bacteria properly

^a^ In 1998, Dr. Sigmund Socransky categorised periodontal pathogens into different colours that are associated with the disease activity of periodontitis.
